# Effects of Tea Polyphenol Palmitate Existing in the Oil Phase on the Stability of Myofibrillar Protein O/W Emulsion

**DOI:** 10.3390/foods11091326

**Published:** 2022-05-02

**Authors:** Jianchao Li, Zongyun Yang, Zhen Li, Ling Wu, Juan Shen, Jinhua Wang, Peng Wang

**Affiliations:** 1 Jiangsu Lihua Animal Husbandry Co., Ltd., Changzhou 213000, China; 15061999761@163.com; 2 Key Laboratory of Meat Processing and Quality Control, MOE, College of Food Science and Technology, Nanjing Agricultural University, Nanjing 210095, China; 2021208021@stu.njau.edu.cn (Z.Y.); 2018808149@njau.edu.cn (Z.L.); 2016027@njau.edu.cn (J.S.); 3 Foshan Shunde Midea Washing Appliances MFG. Co., Ltd., Foshan 528000, China; wuling502014@163.com (L.W.); wangjh12@midea.com (J.W.)

**Keywords:** emulsion, tea polyphenol palmitate, myofibrillar protein, concentration

## Abstract

This study aimed to explore the effect of adding different concentrations (0, 0.01%, 0.03%, and 0.05% (*w*/*w*)) of tea polyphenol palmitate (TPP) in the oil phase on the emulsifying properties of 5 and 10 mg/mL myofibrillar protein (MP). Particle size results revealed that the flocculation of droplets increased as TPP concentration increased and that droplets in 5 mg/mL MP emulsions (25–34 μm) were larger than in 10 mg/mL MP emulsions (14–22 μm). The emulsifying activity index of 5 mg/mL MP emulsions decreased with increasing TPP concentration. The micrographs showed that the droplets of MP emulsions exhibited extensive flocculation at TPP concentrations >0.03%. Compared with 5 mg/mL MP emulsions, 10 mg/mL MP emulsions showed better physical stability and reduced flocculation degree, which coincided with lower delta backscattering intensity (ΔBS) and Turbiscan stability index values. The flow properties of emulsions can be successfully depicted by Ostwald–de Waele models (R^2^ > 0.99). The concentrations of TPP and protein affect the K values of emulsions (*p* < 0.05). Altogether, increased protein concentration in the continuous phase could improve emulsion stability by increasing viscosity, offsetting the adverse effects of TPP to a certain extent. This study is expected to promote the rational application of TPP in protein emulsion products of high quality and acceptability.

## 1. Introduction

In recent years, how to construct healthy fat substitutes has become a research hotspot in the emulsified meat product field. In order to replace animal fat and optimize fatty acid composition, vegetable oils are usually added to emulsion-based meat systems. However, the presence of unsaturated bonds increases the incidence of oxidation of vegetable oils. With the aim of preventing the oxidation of edible vegetable oils, antioxidants are used to delay or prevent the oxidation of the substrate by removing metal ions, scavenging chain-initiating radicals, and/or quenching singlet oxygen [1]. Natural antioxidants and their derivatives have attracted increasing attention due to their widespread and available resources, high efficiency, and possible health benefits [2,3]. Most of the naturally occurring polyphenols, due to their strongly hydrophilic natures, are prevented from binding to a variety of hydrophobic substrates, limiting their antioxidant efficiency in lipid-rich foods [4]. As one of the emerging classes of antioxidants, lipophenols, such as tea polyphenol palmitate (TPP), can dissolve easily in lipids and exhibit stronger antioxidant activity compared with their parent polyphenols [1,4,5,6].

When considering the use of lipophenols in the oil phases of emulsions, the possible influence of lipid-soluble antioxidants on the physical stability of an emulsion cannot be ignored. Emulsions are thermodynamically unstable [7,8] and in general are separated into two phases over a period of time because of their different physicochemical mechanisms, such as gravitational separation, coalescence, flocculation, and Ostwald ripening [9]. With the aim of improving emulsion stability, most studies have focused on the aqueous phase regulation of pH [10,11], ionic strength [12,13], and emulsifier hydrolysis [14,15]. Some researchers have also altered the oily phases with different hydrophobicity [16]. However, the role of oily phase concomitants, such as lipophenols, remains unknown.

For these reasons, this study was conducted to study the effect of addition of TPP to an oil phase on MP emulsion stability. TPP concentration-dependent effects influenced by continuous phase proteins were studied for the first time. This work focused on the combined effects of TPP and myofibrillar protein (MP) concentration on the physical stability of MP–soybean oil emulsion by analyzing the emulsifying activity index (EAI) and emulsion stability index (ESI), droplet size distribution, the ζ-potential of emulsion, viscosity, and microstructural images. Meanwhile, the physical stability (delta backscattering intensity (ΔBS) and Turbiscan stability index (TSI)) was also examined.

## 2. Materials and Methods

### 2.1. Materials

Chicken breasts and Jinlongyu soybean oil (Yihai Kerry Food Company, Shanghai, China) were purchased from a supermarket in Nanjing. TPP was obtained from Shanghai Yuanye Biotechnology Co., Ltd. (Shanghai, China). All the chemicals were of analytical or better grade.

### 2.2. Preparation of MP

MP was extracted according to our previously described method [17]. All extraction steps were strictly operated in a cold storage facility at 0–4 °C. For subsequent testing, the final MP concentration was determined by the biuret method, and bovine serum albumin (BSA) was used as the standard.

### 2.3. Preparation of MP-Stabilized Emulsions

MP was dissolved in 20 mM phosphate-buffered saline (PBS) which contained 0.6 M NaCl (pH 7). The 5 and 10 mg/mL MP solutions were stored at 4 °C until use. To ensure complete dissolution, different concentrations of TPP (0, 0.01%, 0.03%, and 0.05% (*w*/*w*)) were added to the soy oil and heated at 80 °C for 4 min [18]. O/W emulsions stabilized by MP were prepared according to the method of Li [19]. In brief, 10 g of soy oil with TPP and 90 g of MP solution were mixed with a homogenizer (Ultra Turrax T25 BASIS, IKA, Staufen im Breisgau, Germany). The shear emulsification process lasted for 2 min at 10,000 rpm in a cold chamber filled with crushed ice to prevent denaturation caused by excessively high temperatures. Immediately after homogenization, the samples were sealed and stored at 4 °C for analysis within 24 h.

### 2.4. Droplet Surface Average Size (d_3,2_)

The droplet surface average sizes of emulsions were measured according to our previously reported method [19] using a Malvern Mastersizer 3000 (Malvern Instruments Ltd., Malvern, Worcestershire, UK). The refractive indexes of the dispersed phase (soy oil) and dispersant medium were 1.456 and 1.33, respectively. For the indication of stability, fresh O/W emulsions stored at 4 °C were used to evaluate any change in PSD after our treatment.

### 2.5. ζ-Potential Measurements

The ζ-potentials (mV) of the emulsions diluted at a ratio of 1:100 with deionised water were measured according to the reported method [20] by ζ-potential measurement using a Malvern Zetasizer Nano (ZS 90) analyzer (Malvern Instrument Co., Ltd., Worcestershire, UK) and a DTS-7010 capillary cuvette at 25 °C. The samples were diluted 500 times with deionized water.

### 2.6. Measurement of EAI and ESI

The emulsifying activity and emulsifying stability were measured according to a previous study [21], with slight modifications. Briefly, 50 μL aliquot of emulsion was carefully pipetted from the bottom and added to 5 mL of SDS solution (0.1%, *w*/*v*) at 0 and 10 min. The absorbance at 500 nm was measured and SDS solution was used as the blank. The EAI and ESI were evaluated using the following equations:

(1) $$\mathrm{EAI}\left(m^{2}/g\right)=\left(2\times 2.303\times A_{0}\times N\right)/\left(c\times \varphi \times 10000\right)$$

(2) $$\mathrm{ESI}\left(\min\right)=\left(A_{0}/\Delta A\right)\times t$$

where A_0_ represents the absorbance at 500 nm (measured at 0 min), N represents the dilution factor (100), c represents the weight of protein per volume before the emulsion was formed (g/mL), ΔA represents the change in absorbance between 0 min and 10 min (A_0_–A_10_), and t represents the time interval (10 min).

### 2.7. Visual Appearance

Visual appearance was monitored to represent MP emulsion stability. Fresh emulsions were poured into glass bottles and sealed with a cap, and all emulsion samples were stored at 20 °C in the dark until analysis. The creaming stability of the emulsion samples was monitored by visual observation of the height of the aqueous phase formed at the bottom of the bottles. Photographs of the emulsions at the 1st, 3rd, 5th, 7th, and 14th day were captured using a digital camera. Optical micrographs were visualized at 20 °C using a Scope A1 (Carl Zeiss, Gottingen, Germany).

For the obvious separation of emulsions, creaming index (CI) was determined according to the ratio of the heights of the serum layer to the heights of the total emulsion on the days of observation.

### 2.8. Stability Analysis with a Multiple Light Scatterer

TSI was evaluated using a vertical scan analyzer (Turbiscan Tower, Formulation, Toulouse, France) based on our previously reported study [22]. Fresh emulsions were immediately put into a cylindrical cell and scanned into three segments at 25 °C (first every 110 s for 5 h, then every 5 min for 5 h, then every 10 min for 5 h, and finally every 30 min for 15 h). Moreover, the delta data of backscattering intensity (ΔBS) were evaluated on the basis of the method of [23]. ΔBS was also recorded for 30 h by another diode, which subtracted the reference scan (by default, the first one) to the other scans (i.e., using this reference scan as a blank to amplify the variations and visualize them more easily).

### 2.9. Viscosity

The viscosity of emulsions within 24 h was conducted following our previously reported method [17]. The behavior of emulsions can be fitted using the Ostwald–de Waele model, and the model has been reported for meat-production applications [24].

(3) $$\eta_{\mathrm{app}}=K\times \gamma {˙}^{n-1}$$

where η_app_ represents the apparent viscosity (Pa∙s), K represents the consistency index (Pa·s^n^), γ˙ represents the shear rate (s^−1^), and n represents the flow behavior index (dimensionless).

### 2.10. Data Analysis

All experiments were performed at least thrice. Three independent biological repetitions and three technical repetitions were performed. The data are shown as the means ± SD of all of the replicates. Statistical analysis was performed using the SAS software (version 8.0) with one-way ANOVA and Duncan’s multiple range test (SAS Institute Inc., Cary, NC, USA). Differences with a *p*-value <0.05 were regarded as significant.

## 3. Results

### 3.1. Effect of TPP on the Droplet Size Distribution of MP O/W Emulsion

**Figure 1:**
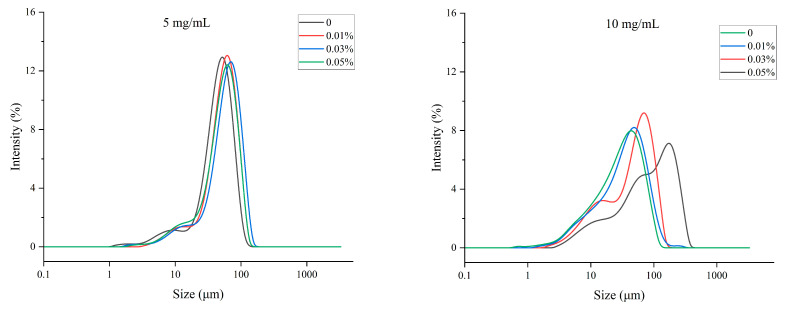
The droplet size distribution of MP O/W emulsion with different concentrations of TPP.

The droplet size of emulsions could be influenced by TPP within the dispersed phase, as shown in Figure 1. From the results depicted in Table 1, the MP emulsion without TPP in the oil phase showed a droplet diameter of approximately 25 μm. The existence of TPP in the oil phase led to the significantly increased droplet diameter of 5 mg/mL MP emulsions, ranging from 31 μm to 34 μm, and 10 mg/mL MP emulsions, ranging from 14 μm to 21 μm. The particle size could increase with the formation of aggregates [25]. Further comparison exhibited that the changing trend of 5 mg/mL and 10 mg/mL MP groups was different. The droplet size of 5 mg/mL MP emulsions was higher than that of 10 mg/mL MP emulsions (*p* < 0.05) with the same TPP concentration. With the same TPP concentration, the droplet size of emulsions could characterize the area of the total O/W interface [26]. The smaller droplet size of emulsions refers to the larger area of the total O/W interface [27], indicating that the emulsifying capacity of emulsions was better under the conditions of the same oil phase [28]. Generally, for protein-stabilized emulsions, the increase in droplet size could be attributed to flocculation and coalescence [29].

### 3.2. Effect of TPP on the ζ-Potential of MP

The changes in the surface charges of the emulsions are reflected in the electrical charge distributions of the layers, providing an efficient method for evaluating the electrostatic interactions between emulsion droplets [30], which could be affected by many factors (salt concentration, pH, and temperature [21,31]. As presented in Table 2, all emulsions had negative ζ-potential values. The emulsion without TPP in the oil phase had a higher absolute ζ-potential value than those with TPP added in the dispersed phase (*p* < 0.05). It has been reported that emulsions with absolute zeta potentials exceeding 20 mV (negative or positive) commonly result in colloidal stability [32]. At the same TPP concentration of 0.01%, the varied effects of ζ-potential values of 5 and 10 mg/mL MP might illustrate that a higher protein concentration (*p* < 0.05) could improve the stability of emulsions. These results indicated that emulsions with TPP had weakened electrostatic repulsion, making them more resistant to further coalescence.

### 3.3. Effect of TPP on the EAI and ESI of MP

The ability of protein to absorb at the O/W interface and to stabilize emulsion droplets could be reflected by the EAI and ESI [33]. Table 3 shows that EAI and ESI can be affected by TPP (*p* < 0.05). The EAI is defined as the total interfacial area of droplets, reflecting emulsification performance or efficiency [34]. ESI values can measure the stability of emulsions over a defined time period [35,36]. The emulsion without TPP in the oil phase had a higher EAI and ESI than those with TPP added in the dispersed phase (*p* < 0.05). No significant difference (*p* > 0.05) in EAIs was recorded between the 0.01% and 0.03% TPP groups. With the increase in TPP concentration to 0.05%, the EAI of emulsion significantly decreased to 0.49, which is only one-third of that of the control group. Compared with the control group, the ESI of emulsion with 0.05% TPP also showed a dramatic decrease (*p* < 0.05). Notably, the lowest ESI value of 10 mg/mL MP emulsions was higher than those of all 5 mg/mL MP emulsions. Moreover, the droplet size of the emulsion measured the EAI and ESI of emulsion, decreased with increasing MP concentration. A similar trend has been reported in a previous study [37]. At lower protein concentrations, the larger diffusion coefficients of MP facilitated the formation of new droplets, indicating the higher EAI of 5 mg/mL MP emulsions. Protein migration in a diffusion-dependent manner could be prevented by the activation energy barrier when the protein concentration was high. A higher protein concentration might reduce the effectiveness of protein adsorption, resulting in the lower EAI of 10 mg/mL MP emulsions.

### 3.4. Effect of TPP on the Appearance of MP O/W Emulsion

#### 3.4.1. Visual Appearance

**Figure 2:**
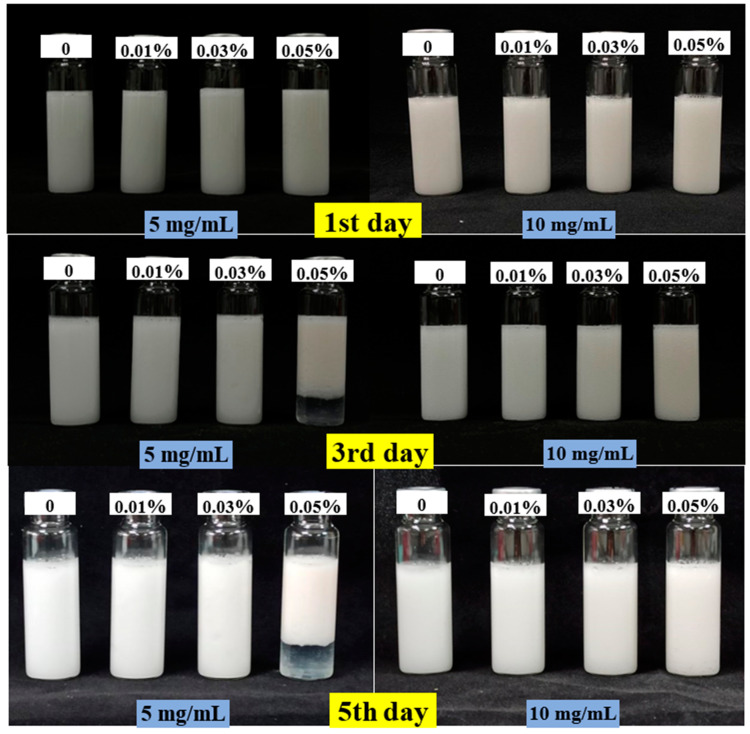

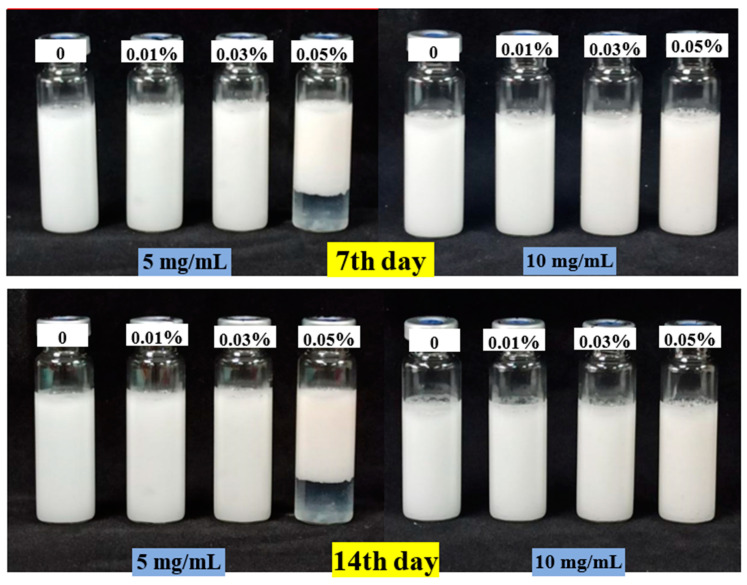
The visual appearance of MP O/W emulsions with different concentrations of TPP.

The visual appearance of emulsions was evaluated immediately after their preparation. On the first day, all emulsions demonstrated a creamy, milky appearance and were visually opaque, as shown in Figure 2. For both the 5 mg/mL and 10 mg/mL MP groups, the visual appearance of the control, 0.01%, and 0.03 TPP groups remained uniform and stable. However, the 5 mg/mL MP emulsion sample with 0.05% TPP developed a creaming layer after 3 days of storage. Creaming behavior was not detected in a layer of aqueous phase with the absence of TPP from emulsions or in higher protein emulsions. More droplet flocculation in 5 mg/mL MP emulsions with 0.05% TPP caused the larger size relative to the 10 mg/mL MP emulsions. Furthermore, the CI of obvious separation of emulsions (5 mg/mL MP emulsions with 0.05% TPP) increased from 22.9% (3rd day) to 30.58% (14th day). At the same TPP concentration of 0.05%, the varied effects of 5 and 10 mg/mL MP might illustrate that the higher protein concentration formed the thicker continuous phase. Therefore, the higher protein concentration could prevent creaming, coalescence, and aggregation during 14 days of storage and improve the stability [30].

#### 3.4.2. Optical Micrographs

**Figure 3:**
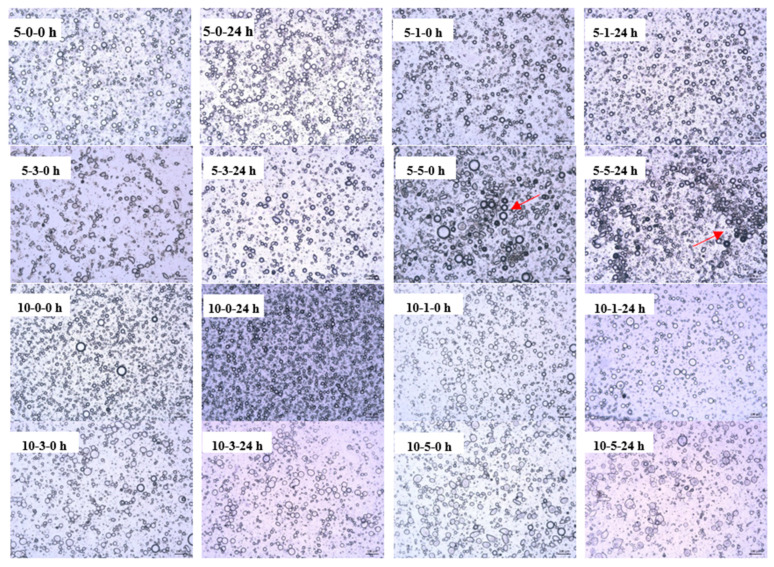
The micrographs of MP O/W emulsion with different concentration of TPP at 0 and 24 h. The droplets appeared to aggregate significantly at high concentrations of TPP (arrow).

The optical micrographs of the systems were also determined within 24 h of establishing the emulsion at ambient temperature (Figure 3). Particle size distribution and shape are crucial for emulsions stabilized by proteins [38]. For unstable emulsions, changes in particle sizes or flocculation were observed. The visual appearance of emulsions changed at TPP doses >0.03%. Oil droplet distribution was relatively even in emulsions containing 0% and 0.01% TPP, and extensive flocculation of emulsion droplets appeared in the presence of 0.03% and 0.05% TPP. The oil droplets were non-uniformly dispersed with aggregates in the emulsions at 5 mg/mL MP with TPP. However, the 10 mg/mL MP emulsions appeared to be more homogeneous than 5 mg/mL MP emulsions. The size of the emulsion increased slightly at 24 h (Figure 3). The optical microscopy of the emulsions with 0.03% TPP indicated that the emulsions had the same characteristics at 0 and 24 h. There was less sign of flocculation with high protein concentrations [37].

### 3.5. Effect of TPP on the Backscattered Light Properties of MP O/W Emulsion

**Figure 4:**
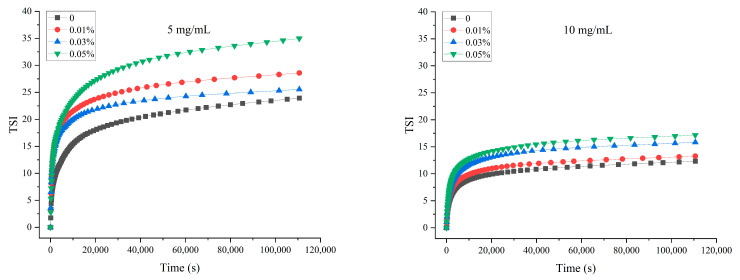
The TSI of MP O/W emulsion with different concentrations of TPP during 30 h.

Based on difference curve calculations of backscattered light, optical characterization of stability by TSI provides valuable information on the creaming kinetics of emulsions. For both 5 and 10 mg/mL MP groups, the TSI of all emulsion groups increased and reached a plateau over time (Figure 4). TPP existing in the oil phase had detrimental effects on the stability of emulsions, and 0.05 TPP had the worst effect, as evidenced by the highest TSI value. All emulsions with 5 mg/mL of MP as the emulsifier exhibited a higher tendency to creaming compared with the corresponding TPP treatments of 10 mg/mL MP emulsion.

**Figure 5:**
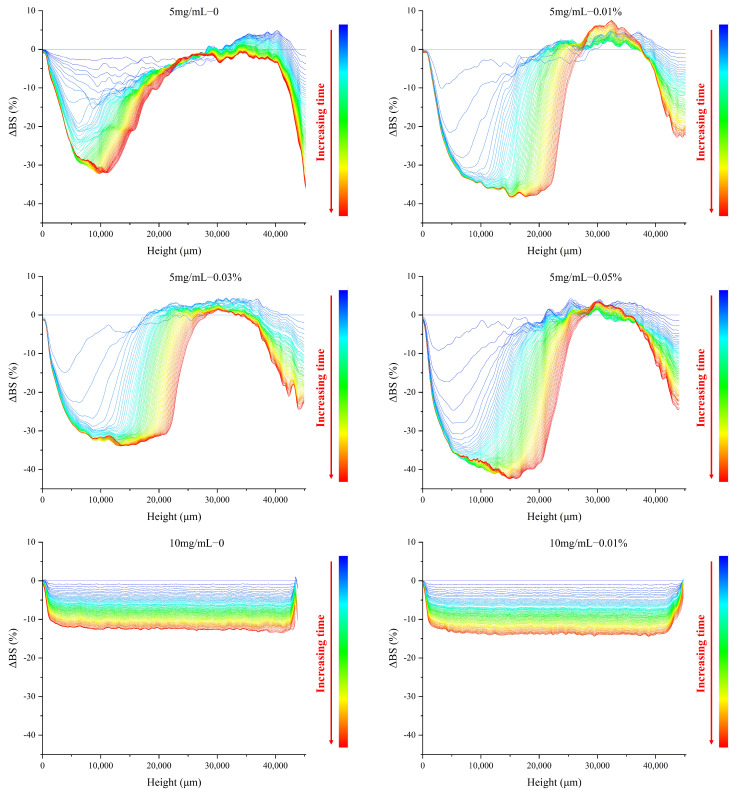

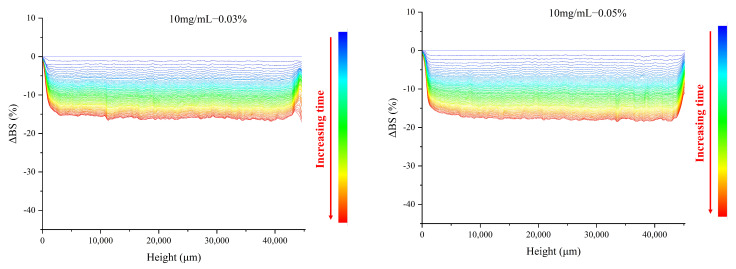
The ΔBS of MP O/W emulsions with different concentrations of TPP during 30 h.

Furthermore, with the high curve slope and values of TSI, segregative phase separation occurred, which could be consistent with the results for the ΔBS profiles (Figure 5). The global and local changes in the stability of the emulsions could be better reflected by the dynamics of BS [39]. The droplets moved to the top of the system to form a creaming layer during the phase separation, and the ΔBS of this system could access this information as a function of height. The blue and red curves represent the signals that were detected within 30 min and 15–30 h, respectively. The ΔBS values of 5 mg/mL MP emulsions were relatively higher than those of 10 mg/mL MP emulsions, indicating that lower continuous phase protein concentration is associated with higher levels of instability. This instability is owed to the Brownian motion of particles/droplets; at lower protein concentrations, the viscosity of the continuous phase is reduced, resulting in the free movement of emulsion droplets and causing aggravating phase separation and variation in ΔBS values at different heights from the bottom.

The samples with TPP existing in the oil phase exhibited higher values of ΔBS, which indicated that the emulsions had higher instability. The polarity differences of oil phases upon the addition of TPP could have induced this instability, as discussed in the previous section.

### 3.6. Effect of TPP on the Viscosity of MP Solution and O/W Emulsion

**Figure 6:**
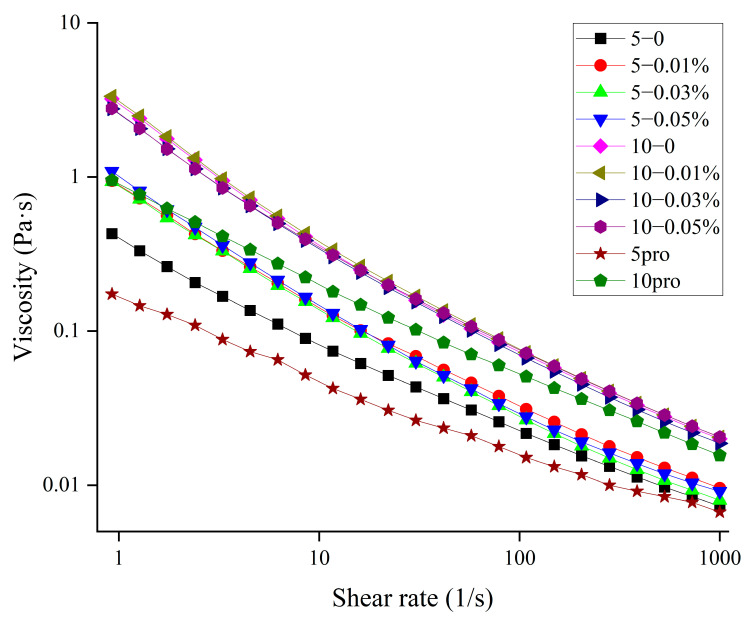
The viscosity of MP solution and O/W emulsion with different concentration of TPP.

Another study also found that increasing the viscosity or forming a gel network can be beneficial to hinder the movement of solution particles [40]. The results for the viscosity (η) of MP solutions and O/W emulsions are shown in Figure 6. The apparent viscosity of protein solutions and emulsions all demonstrated a decreasing trend with increasing shear rates, indicating that all emulsions exhibited a shear-thinning behavior, which has been reported in meat emulsion systems stabilized by proteins [17,41]. There was a strong correlation between the apparent viscosity of emulsions and emulsion stability [42]. The continuous increase in MP concentration caused the emulsion viscosity to increase. A highly viscoelastic interfacial film would be formed to prevent flocculation [43,44]. According to the Stokes equation, emulsion stability could be improved by increasing the viscosity [43]. The consistency coefficient (K) and flow index (*n*) obtained from the Ostwald–de Waele model were summarized (Table 4). The Ostwald–de Waele model was found to be an adequate model (R^2^ > 0.99) to illustrate the flow behavior of MP emulsions. The n value, which determined the deviation from Newtonian flow [45], was less than 1 (see Table 3), indicating that all the MP emulsions were pseudoplastic fluids [46]. These results coincided with the obvious effects of MP concentrate on the K and n values (*p* < 0.05). A slight increase (*p* < 0.05) in the *n* values of emulsions with more than 0.03% TPP indicated that the shear-thinning behavior of emulsions was restrained [20]. However, the *n* values showed a marked decrease in 5 mg/mL emulsions to which TPP was added, whereas they increased for 10 mg/mL emulsions. The same trend was observed for the K values of emulsions. The K values of 5 mg/mL emulsions increased, whereas those of 10 mg/mL emulsions decreased. These results indicated that increasing the viscosity of the continuous phase could improve emulsion stability.

## 4. Conclusions

In this study, the effects of TPP on the emulsions stabilized by different MP concentrations were studied. The results showed that TPP addition had limited effects on droplet size and emulsion stability, whereas the concentration of MP solutions increased emulsion stability by increasing the viscosity of the continuous phase. Compared with 5 mg/mL MP emulsions, 10 mg/mL MP emulsions showed smaller droplet sizes and better physical stabilities, which coincided with ΔBS values and lower TSI values. The variation range of ΔBS for the emulsion stabilized by 10 mg/mL MP without TPP was the smallest, and the TSI value of this group was the lowest. It also showed better storage stability (14 days). Additionally, no more than 0.03% (*w*/*w*) TPP can be accepted, as evidenced by no extensive flocculation of emulsion droplets though the micrographs. All the emulsions showed shear-thinning behavior. As the concentration of MP increased, the K values of emulsions decreased and the n values increased. This study demonstrates that increasing protein concentration in the continuous phase could offset the adverse effects of adding TPP to emulsions and has provided new ideas for the application of TPP. Our research will encourage efforts to exploit the potential of emulsion manipulation mediated by TPP. Emulsifying properties can be influenced by ionic strength, pH, and temperature, etc. Future research on the above parameters would be valuable for better insight into TPP behavior with respect to emulsion physical stability.

## Figures and Tables

**Table 1:** Effects of TPP on droplet size (d_3,2_) of MP O/W emulsions.

d_3,2_ (μm)	0	0.01%	0.03%	0.05%
5 mg/mL	25.25 ± 0.14 ^Ac^	34.5 ± 1.90 ^Aa^	34.82 ± 1.13 ^Aa^	31.62 ± 0.58 ^Ab^
10 mg/mL	14.12 ± 0.80 ^Ba^	16.55 ± 1.56 ^Bb^	17.03 ± 0.84 ^Bb^	21.68 ± 0.96 ^Bc^

Notes: Values are means ± SD. ^A,B^ These values indicate significant differences between the various protein concentrations under the same TPP concentration (*p* < 0.05). ^a–c^ These values indicate significant differences for the various TPP concentrations under the same protein concentration (*p* < 0.05).

**Table 2:** Effects of TPP on the ζ-potential of MP O/W emulsions.

ζ-Potential (mV)	0	0.01%	0.03%	0.05%
5 mg/mL	−20.75 ± 0.7 ^Ab^	−19.96 ± 0.16 ^Ab^	−19.98 ± 0.17 ^Ab^	−18.62 ± 0.7 ^Aa^
10 mg/mL	−21.97 ± 0.23 ^Bd^	−20.84 ± 0.13 ^Bc^	−20.00 ± 0.36 ^A^^b^	−18.99 ± 0.13 ^Aa^

Notes: Values are means ± SD. ^A,B^ These values indicate significant differences between the various protein concentrations under the same TPP concentration (*p* < 0.05). ^a–c^ These values indicate significant differences for the various TPP concentrations under the same protein concentration (*p* < 0.05).

**Table 3:** Effects of TPP on EAI and ESI of MP O/W emulsions.

Treatment		0%	0.01%	0.03%	0.05%
5 mg/mL	EAI (m^2^/g)	1.67 ± 0.095 ^Aa^	0.83 ± 0.087 ^Ab^	0.75 ± 0.048 ^Ab^	0.49 ± 0.059 ^Ac^
	ESI (min)	62.91 ± 2.52 ^Aa^	48.69 ± 3.96 ^Ab^	28.93 ± 7.09 ^Bc^	15.04 ± 1.71 ^Bd^
10 mg/mL	EAI (m^2^/g)	1.32 ± 0.17 ^Ba^	0.94 ± 0.09 ^Ab^	0.42 ± 0.07 ^Bc^	0.28 ± 0.01 ^Bc^
	ESI (min)	28.68 ± 4.74 ^Bb^	31.74 ± 1.71 ^Bb^	57.68 ± 11.94 ^Aa^	53.31 ± 5.81 ^Aa^

Notes: Values are means ± SD. ^A,B^ These values indicate significant differences between the various protein concentrations under the same TPP concentration in ESI/EAI values (*p* < 0.05). ^a–c^ These values indicate significant differences for the various TPP concentrations under the same protein concentration (*p* < 0.05).

**Table 4:** Effects of TPP on EAI and ESI of MP O/W emulsions.

Treatment (C_TPP_, *w/w*)		0	0.01%	0.03%	0.05%
5 mg/mL	K	0.46 ± 0.042 ^Bb^	0.60 ± 0.088 ^Ba^	0.64 ± 0.10 ^Ba^	0.70 ± 0.042 ^Ba^
	n	0.30 ± 0.012 ^Aa^	0.29 ± 0.024 ^Aab^	0.27 ± 0.041 ^Aab^	0.24 ± 0.01 ^Ab^
10 mg/mL	K	3.06 ± 0.098 ^Aa^	2.99 ± 0.081 ^Aab^	2.84 ± 0.057 ^Ab^	2.82 ± 0.11 ^Ab^
	n	0.083 ± 0.027 ^Ba^	0.076 ± 0.0019 ^Bab^	0.10 ± 0.0073 ^Ba^	0.099 ± 0.0079 ^Ba^

Notes: Values are means ± SD. ^A,B^ These values indicate significant differences between the various protein concentrations under the same TPP concentration in K/n values (*p* < 0.05). ^a–c^ These values indicate significant differences for the various TPP concentrations under the same protein concentration (*p* < 0.05).

## Data Availability

The data presented in this study are available on request from the corresponding author. The data are not publicly available due to the whole experiment is still proceeding.
